# Mortality analysis of captive red panda cubs within Chengdu, China

**DOI:** 10.1186/s12917-022-03170-2

**Published:** 2022-02-10

**Authors:** Songrui Liu, Yunli Li, Dongsheng Zhang, Xiaoyan Su, Chanjuan Yue, James E.Ayala, Xia Yan, Rong Hou, Lin Li, Yi Xie, Guifu Zhuo, Rita McManamon, Kuixing Yang

**Affiliations:** 1 Chengdu Research Base of Giant Panda Breeding, Sichuan Key Laboratory of Conservation Biology for Endangered Wildlife, Chenghua District, 1375 Panda Road, Chengdu, 610081 Sichuan China; 2 Zoo and Exotic Animal Pathology Service, Infectious Diseases Laboratory, Department of Small Animal Medicine and Surgery Department of Pathology College of Veterinary Medicine, University of Georgia, Athens, GA 30602 USA

**Keywords:** China, Cub mortality, *Ex-situ* conservation, Pathological characteristics, Red panda

## Abstract

**Background:**

The red panda has been classified as an endangered species due to the decreased number in the world and disease is considered as a great threat to the health and survival of the cubs in captivity.

**Results:**

This study analyzed 32 red panda cub mortalities (15 females and 17 males, age less than two months) through gross necropsy, microbiological examination, and histopathological observation at the Chengdu Research Base of Giant Panda Breeding, China, during 2014–2020. The results showed that screenings for canine distemper virus, canine parvovirus, rotavirus and parasite infection were all negative, however bacteria such as *Klebsiella pneumoniae*, *Proteus mirabilis*, *Escherichia coli*, *Enterococcus faecalis*, *Pseudomonas* were isolated from the tissue samples of some cubs. The major causes of death were respiratory (43.75%) and digestive system disease (28.13%), followed by cardiovascular disease (12.5%) and neonatal stillbirths (9.38%). Renal system diseases and trauma were also detected, at lower incidence (one case for each). The mortality rate within 15 days of birth was 68.75% and gradually decreased with age, there was no significant difference in gender.

**Conclusion:**

This study can provide a scientific basis for the analysis of the cause of death among red panda cubs in captivity, so as to improve the survival rate, help build the captive population and further the *ex-situ* conservation management of this endangered species. Additionally, our research may also provide insights into the *in-situ* conservation of wild red pandas by identifying emerging disease threats within the wild population and potential treatment for rescued individuals.

## Background

The red panda is the only extant member of the Ailuridae family, and is divided into two subspecies, *Ailurus fulgens fulgens* and *Ailurus fulgens styani* [1]. Recently, genetic studies have confirmed that individuals may be categorized into two distinct species: the Himalayan red panda (*Ailurus fulgens*) and the Chinese red panda (*Ailurus styani*), with the two species divided by the Yalu Zangbu River [2].

The red panda has been classified as an endangered species by the International Union for the Conservation of Nature (IUCN). Although the wild population is difficult to survey, it is believed to have decreased to less than 10,000 mature individuals due to deforestation, habitat loss, poaching and disease [3]. Mortalities caused by disease are also an important factor in the *ex-situ* population in China [4]. Captive newborn and adult red panda mortalities are mainly caused by infectious diseases, as well as gastrointestinal, circulatory, respiratory and renal diseases, and neoplasia [5]. Canine distemper virus (CDV) is the major cause of death in the *ex-situ* population within China [4], with other bacterial diseases also contributing to red panda mortalities [6, 7]. The researchers have investigated 10 red panda mortalities at the Chengdu Research Base of Giant Panda Breeding (CRBGPB) in 2006 and found that heartworm infection was the direct cause of the death for these animals [8].

The red panda is a seasonally breeding animal, both males and females are sexually mature at the age of 18–20 months. The breeding season is from mid-January to mid-March each year, and the birth season is from June to August. In captivity, the rate of natural mating and conception of red pandas is relatively high, but research has shown that the low survival rate of cubs (25%-76.26%) has become the main problem restricting the growth of the captive population [9, 10]. Previous research investigated 201 red panda mortalities from 1994 to 2006 and found 59% of red pandas died within 1 year after birth, and 35% of red pandas died within 3 days of birth [11]. The study of the death of 530 red pandas in North America between 1992 and 2012 showed that cub mortality was as high as 40.2% [12]. To date, there are scant research reports about the causes of death of captive red panda cubs in China.

The CRBGPB currently has 163 captive red pandas (*Ailurus fulgens styani)* and is one of the main institutions in China for the *ex-situ* conservation of the species. In order to better understand factors underlying red panda cub mortalities (less than two months of age), we collected samples from 32 red panda cubs that died at the CRBGPB between 2014 and 2020 and performed gross necropsy, microbiological examination, and histopathological observations as part of routine animal management. These results provide a scientific basis for current and future management strategies, for the health and survival of red pandas cubs and suggestions for improving the *ex-situ* management of the species in China.

## Results

### Microbiological examination

Within 32 red panda cub mortalities between 2014 and 2020, CDV, canine parvovirus (CPV), and rotavirus (RV) antigens were negative in all cases. No parasitic infections were detected. In several cases, bacteria such as *Klebsiella pneumoniae*, *Proteus mirabilis*, *Escherichia coli*, *Enterococcus faecalis*, *Pseudomonas* were isolated from tissue samples.

### Gross necropsy and histopathological observation

The primary cause of death for each cub was identified by gross necropsy, histopathological observation and microbiological examination. We divided preliminary causes of death into the following categories: respiratory disease, digestive disease, cardiovascular disease, renal disease, stillborn and trauma (Table 1). The mortality rate within 15 days of birth was 68.75%, mortality reduced to 21.87% (16–30 days) and 9.38% (31–60 days) of age. There was no significant difference between genders.

**Table 1:** Primary cause of death in red panda cubs at Chengdu during 2014–2020

Primary cause of death	Age at death			Gender		Total	
	0-15 days	16-30 days	31–60 days	Female	Male	Number	Ratio(%)
Respiratory Disease	6/22	5/7	3/3	7/15	7/17	14	43.75%
Digestive Disease	7/22	2/7	0	4/15	5/17	9	28.13%
Cardiovascular Disease	4/22	0	0	2/15	2/17	4	12.50%
Stillborn	3/22	0	0	1/15	2/17	3	9.38%
Renal Disease	1/22	0	0	0	1/17	1	3.13%
Trauma	1/22	0	0	1/15	0	1	3.13%
Total	22	7	3	15	17	32	100%

**Fig. 1:**
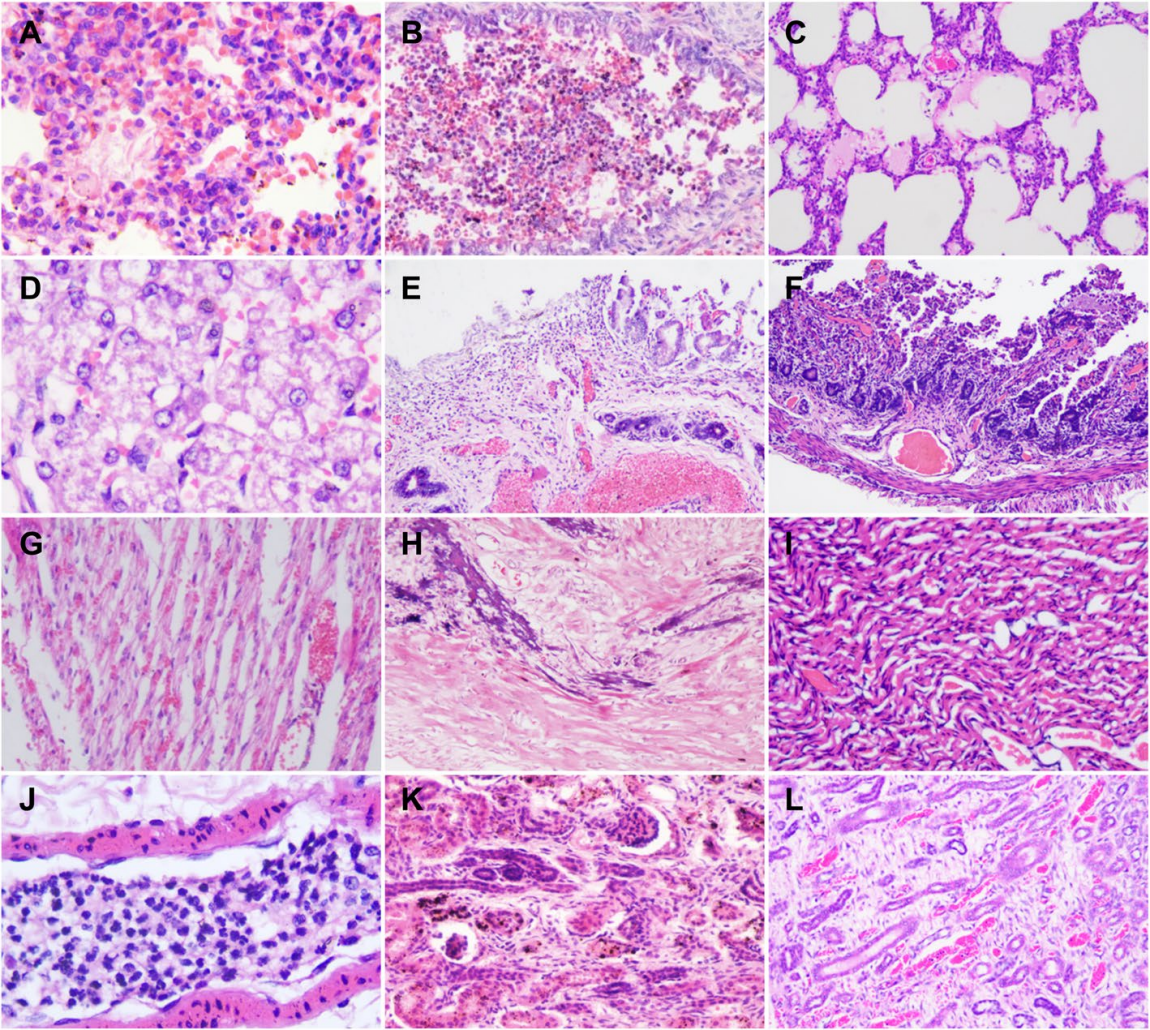
Histopathological observations of the tissue samples from red panda cubs in the Chengdu Research Base of Giant Breeding, Sichuan Province, P.R. China, 2014–2020. Hematoxylin and eosin (HE). **A** Typical acute interstitial pneumonia with thickened alveolar septa and mononuclear cell infiltration, 200 × . **B** Lobar bronchopneumonia, the alveolar wall is congested and thickened, and the bronchial lumen is filled with numerous neutrophils, red blood cells and a variable number of exfoliated epithelial cells, 200 × . **C** Acute fibrinous pneumonia with large amounts of plasma and exfoliated epithelial cells in the alveolar cavity, 100 × . **D** Hepatocellular hydropic degeneration and steatosis, 400 × . **E** Gastric mucosal epithelial necrosis with lymphoplasmacytic inflammation, 100 × . **F** Duodenum mucosal necrosis with hemorrhage and inflammation, 100 × . **G** Widened intermyocardial edema, leaky hemorrhage and granular degeneration, 100 × . **H** Myocardial necrosis and calcification, 100 × . **I** Intermuscular congestion and extensive fatty degeneration of cardiomyocytes, 200 × . **J** Pulmonary vein neutrophilic thrombosis, 400 × . **K** Renal cortical glomerular and tubular loss, with replacement interstitial fibrosis, 200 × . **L** Renal cortical tubular necrosis, interstitial fibrosis and edema, 100 ×

Respiratory disease was the primary cause of death in 14 cases, six died during the first 15 days postpartum. Grossly, a large amount of pleural effusion was seen during necropsy, the lungs were bleeding, congested, and mottles in color, with pinkish white and dark red interlaced. Food residue was found in the trachea in one case in which the cub was hand-reared. Histopathology showed acute interstitial pneumonia (Fig. 1A), lobar bronchopneumonia (Fig. 1B), and acute fibrinous pneumonia (Fig. 1C), with extensive alveolar wall thickening, inflammatory cell infiltration, necrosis and shedding of alveolar epithelial cells.

Digestive system diseases were also important. Nine cubs had serious digestive tract injuries. A considerable amount of black contents was found in the esophagus, the liver was dark red and slightly swollen, the stomach was full and bloated, the intestinal wall was thin, the mesenteric hemorrhage, and the intestinal contents were not formed. Histopathological observation showed hepatocellular hydropic degeneration and steatosis (Fig. 1D), gastric mucosal epithelial necrosis with lymphoplasmacytic inflammation (Fig. 1E), duodenum mucosal necrosis with hemorrhage and inflammation (Fig. 1F).

Pericardial effusion, enlarged volume, pale heart apex, atrial congestion, hard texture, and vascular congestion were observed during necropsy in cubs which died due to cardiovascular diseases. Histopathological observations typically showed widened intermyocardial edema, leaky hemorrhage and granular degeneration (Fig. 1G), myocardial necrosis and calcification (Fig. 1H), and intermuscular congestion and extensive fatty degeneration of cardiomyocytes (Fig. 1I). Thrombosis was seen in some pulmonary veins, and some cases showed neutrophilic inflammation (myocarditis) (Fig. 1J).

For the cub which died from renal disease, the bilateral kidneys were uneven in size, with hard texture and hyperemia on the surface. Histopathological observations showed that renal cortical glomerular and tubular loss, with replacement interstitial fibrosis (Fig. 1K), renal cortical tubular necrosis, interstitial fibrosis and edema was also identified (Fig. 1L).

## Discussion

Red pandas are seasonal breeders with estrus occurring from mid-January to mid-March each year. The gestation period ranges between 114 and 145 days, with 90% of the births occurring in June and July [1]. The first few days of life for red panda cubs is a critical period, a previous 12-year study showed that 118/201 (59%) of red panda cubs died within the first month of life [11]. In this study, 29 of the 32 cubs died shortly after birth during the summer. Chengdu is located in the southwest of China, and the temperature and humidity are relatively high during the summer. Although females are susceptible to the high temperature and humidity postpartum, they are sensitive to any disturbance, and there is a high risk that any intervention at the nest box – including examination or manipulation of young—may result in accidental injury to the cubs, as nursing females will frequently attempt to move cubs away from disturbance. Since high temperatures and humidity may also increase the threat of bacterial and fungal infections, all facilities must attend to environmental conditions, including potential monitoring, air conditioning and ensuring close observations of behavior in light of environmental parameters.

Of the recorded mortalities, 43.75% of the cubs (14/32) died from respiratory organ damage, mainly manifested as pulmonary congestion, lobar bronchopneumonia, acute fibrinous pneumonia. This conclusion is consistent with the published analyses of mortality in captive red pandas in North America [12], and in Europe [11]. Previous publications have shown that of causation in lung infections in red pandas include parasitic pneumonia [6], *Pasteurella* [13]*, Staphylococcus aureus* [14] and *Klebsiella pneumoniae* [15]*. Klebsiella pneumoniae* is an important gram-negative opportunistic pathogen that causes pneumonia and other respiratory damage in red panda [15] and giant panda [16], when there are reductions in body condition and immune function. In this study, parasitic infections were not found, but *Klebsiella pneumoniae* was isolated from lung and swabs in one case, *Proteus mirabilis* was isolated from kidney, liver and urine samples in three cases. Previous research also suggests improving the management of daily feeding important to prevent respiratory disease caused by bacterial infection [14–16].

Digestive system diseases are another main cause of mortality. In this study, nine cubs died of digestive system diseases, mainly manifested as necrotizing enteritis or enterocolitis. Previous reports of the main digestive diseases of red panda include parasites [17], gastroenteritis [18], purulent esophagitis [19], viral infections such as canine parvovirus [20], bacterial infections such as *Escherichia coli* [21] and *Proteus mirabilis* [22]*.* In these nine cases, no incidences of parasitism nor viral causation were detected. Although final diagnoses were not always determined for each animal, experience shows that the gastrointestinal tract of red panda cubs is not fully developed, so the principle of “less is better than more” should be adopted in hand-raising cubs, to avoid causing hypermotility and indigestion due to overfeeding.

In this study, 4 cubs died of circulatory system diseases, which manifested as inflammatory and degenerative heart damage. In these 4 cases, the findings were also accompanied by other signs indicating systemic bacterial disease, such as splenitis, hepatitis, and fibrinous pneumonia. some individuals showed clinical symptoms of systemic hypoxia. In these cases, no viral etiology was identified, and *Proteus mirabilis* was isolated from the heart in one case.

During the period covered in this study there were 3 stillborn cubs (9.38%). To increase the reproductive rate of the red pandas, the CRBGPB improved the environmental conditions of captive red panda in 2011. Through these improvements, the reproductive rate improved and the mortality rate of red panda cubs significantly decreased from 44. 5% to 23.74% [10]. However, additional efforts are continuing, to further reduce mortality.

The proportion of red panda cub urinary system infection and trauma in this population was low (*n* = 1), but there are other reports of fatal renal disease in cubs. In our experience, our team documented a single case of *Escherichia coli* -associated pyelonephritis in an adult captive red panda [7].

Based on these findings, early health monitoring of potential breeding females before the breeding season seems advisable, so as to reduce human interference and stress during pregnancy and prior to parturition. Experience with CRBGPB hand-raising of cubs has also proven that intervention with maternal care should be avoided as much as possible, except in cases of maternal abandonment or insufficient production of milk. Since red pandas are highly susceptible to CDV, all individual animals within the breeding plan should be assessed for health, including viral tests, prior to and after breeding season. Vaccination is the best way for CDV prevention, all adult females and males should finish the vaccination procedures before breeding season. Furthermore, other publications have emphasized daily husbandry management techniques such as positive reinforcement training and environmental enrichment, in order to reduce behavioral stressors and stereotypic behaviors, and to promote reproductive ability [23]. A combination of these factors will thereby improve animal welfare and ensure the healthy development of the captive red panda population, so as to provide healthy populations for future reintroduction, and also provide technical support for wild red panda rescue.

## Conclusion

Overall, this study analyzed the causes of death of 32 captive red panda cubs through gross necropsy, microbiological examination, and histopathological observation. This is a retrospective study, encompassing a significant number of years with various levels of final diagnostic findings, this survey provides key information to guide caretakers of neonatal and young pandas. However, many specific pathogens that caused the mortalities still need to be investigated further.

## Methods

### Study area and animals

The CRBGPB (30° 44′22. 80″N, 104°08′28. 64″E) is located at 1375# Panda Road, Northern Suburb, Chenghua District, Chengdu City, Sichuan Province, P.R. China. Covering an area of 66.67 hectares, the CRBGPB serves as an internationally renowned leading center for *ex-situ* conservation of both giant pandas and red pandas, and includes a scientific research and breeding base, a conservation education center, and an educational tourism park. As of 2021, the CRBGPB manages 163 red pandas and has been breeding red pandas since its founding in 1987. It currently houses the largest captive population of red pandas in the world. On 28 April 2012, the CRBGPB has established the Red Panda Conservation and Research Center of Sichuan Provincial Forestry Department. We provided bamboo, bamboo shoots, apples, panda bread like supplement produced at the CRBGPB to the red pandas, while water is provided by artificial streams in the enclosure and water bowls in doors [10, 24].

Routine necropsies on 32 captive red panda cubs were performed between 2014 and 2020. All procedures adhered to Chinese Association of Zoological Gardens (CAZG) guidelines, and the regulations of China. All the adult red pandas receive Purevax® Ferret Distemper (Distemper Vaccine) and NOBIVAC® Rabies (Rabies Vaccine) once a year. We divided the cub mortalities into three groups according to the life stage within the first 2 months: 0-15 days, 16-30 days, and 30-60 days.

### Gross necropsy

The veterinary and research staff of the CRBGPB conducted the necropsies and collected the following data from all cases: date and location, sex, date of birth, coat condition, nutritional status, skin integrity, fractures and other traumatic injuries, condition of the orifices (mouth, eyes, nose, ear, anus, external genitals). Oral swabs, nasal swabs and fecal swabs were collected for the further microbiological examination. In all cases, the vital organs were examined grossly and tissue samples were collected. We also took the photographs of external features during necropsy and assessed gross and histopathological reports retrospectively.

### Microbiological examination

Bacterial culture: Sterilizing the surface of the organs with a scorching scalpel, inserted the inoculation loop into the tissue and collected the samples after aseptic incision, streaked the inoculation loop on the brain heart infusion agar plate, placed the plate in a constant temperature incubator at 37 °C for 12–24 h. Bacteria growth was observed and a typical single colony was selected for further purification, and then identified by Gram stain and 16S ribosomal ribonucleic acid (rRNA) sequencing.

Parasite screening: Collected feces and anal swabs and diluted with water, using centrifugal sedimentation to conduct fecal parasite screening.

Virus detection: Collected oral swabs, fecal swabs and nasal swabs and screened all the samples using polymerase chain reaction (PCR) test for CDV, CPV and RV. The PCR test protocol was improved and optimized in our lab according to previously published techniques [25–27].

### Histopathological observation

At the time of necropsy, we collected a range of tissue samples (heart, lung, liver, kidney, spleen, stomach, intestine, lymph nodes) for histopathology, fixed in 4% paraformaldehyde, embedded in paraffin, sectioned at 5 μm and stained with hematoxylin and eosin (HE). A Leica microscopic imaging system was used to take pictures and record the characteristic histopathological changes.

## Data Availability

The data involving in the manuscript can be obtained from the corresponding author upon reasonable request.

## Abbreviations

IUCN: International Union for Conservation of Nature
CRBGPB: Chengdu Research Base of Giant Panda Breeding
PCR: Polymerase Chain Reaction
CDV: Canine Distemper Virus
CPV: Canine Parvovirus
RV: Rotavirus
CAZG: Chinese Association of Zoological Gardens
rRNA: Ribosomal Ribonucleic Acid
HE: Hematoxylin and Eosin
